# Correlations between particulate matter pollutant factors and morbimortality in Romania—a study with focus on respiratory diseases

**DOI:** 10.3389/fpubh.2026.1819542

**Published:** 2026-06-04

**Authors:** Dragos-Cosmin Zaharia, Alexandru-Nicolae Dimache, Alexandra-Maria Cristea, Stefan Leu, Cristina Jitariu, Irina Ruxandra Strambu

**Affiliations:** 1 Discipline Pneumophthisiology 3, Universitatea de Medicina si Farmacie Carol Davila din Bucuresti, Bucharest, Romania; 2 Department Pneumophthisiology 7, Institutul de Pneumoftiziologie Marius Nasta, Bucharest, Romania; 3 Department of Hydraulics, Sanitary Engineering and Environmental Protection, Universitatea Tehnica de Constructii Bucuresti, Bucharest, Romania; 4 Institutul National de Sanatate Publica, Bucharest, Romania

**Keywords:** lung diseases, morbidity, particulate matter, PM_10_-_2.5_, pollution, public health

## Abstract

Atmospheric pollution greatly impacts public health, since accumulating scientific data found credible evidence between pollutant exposure and various causes of morbimortality. In the 42 counties of Romania some pollutant substances and particles are monitored trough 152 stations. The study focuses on particulate matter (PM_10_ and PM_2.5_) data analysis in correlation with public health statistics. Spearman tests were used to correlate contemporaneous yearly average PM values to morbidity and mortality and showed a statistically significant weak positive correlation between PM_10_ and PM_2.5_ average yearly concentrations (μg/m3) and morbidities for lung cancer (LC) (*r* = 0.211, *p* < 0.001; *r* = 0.166, *p* = 0.022, respectively) and asthma (*r* = 0.264, *p* < 0.001; *r* = 0.31, *p* < 0.001, respectively). For mortalities, PM_2.5_ showed a positive weak correlation with congenital and chromosomal anomalies (CCA) (*r* = 0.21, *p* = 0.004) while PM_10_ showed weaker positive correlations with multiple mortality causes (tuberculosis, CCA, early childhood mortalities (< 27 days, < 1 year and < 5 years). Other correlation results involving morbidities or mortalities from other causes were not reliable due to the high impact of SARS-Cov2 pandemic on hospital admissions and deaths but also on data reporting. The Welch 2-sample T-test performed for the comparison of the most (Gorj, 30.03 μg/m^3^) and least (Harghita, 16.80 μg/m^3^) PM_10_ polluted counties showed an increase in LC (*p* = 0.002) and asthma (*p* = 0.279) admissions for the polluted county. The 3-year exposure window emerged as a predictor for morbidity for lung cancer and asthma using single and dual linear regression models. For asthma, the fine fraction (PM_2.5_) remained the primary independent driver, adding 5.28 admissions per 100,000 inhabitants for every increase with 1 μg/m^3^. For lung cancer, the coarse particle fraction exhibited the strongest independent effect on hospitalizations, adding 2.85 admissions per 100,000 inhabitants for every unit increase in concentration. Based on the results of the linear regression models, avoidable burden maps for morbidity from lung cancer and asthma were built, which can be useful tools for adapting public health and environmental policies on the dynamic of the PM concentrations in time and space.

## 1 Introduction

Air pollution has a substantial impact on the burden of chronic lung disease on public health systems. Pollutants such as particulate matter (PM_10_, PM_2.5_), inorganic substances (NO_2_, SO_2_) or organic substances have various effects on morbidity and mortality. Climate changes also have a contribution influencing the air concentration and equilibrium of these substances in the ecosystem (1).

There are many studies that showed associations between short-term exposure to PM_2.5_ and NO_2_ and morbimortality. For example, a study performed on the data of 135 US cities showed there is a dose-effect relationship between the concentration of PM_2.5_ and mortality as there was a 1.5% increase in all-cause mortality per 10 μg/m^3^ increased in PM_2.5_ concentration (2). A dose-effect relationship between NO_2_ concentration and morbimortality was noted in a 2015 meta-analysis as for a 10 μg/m^3^ increase in 24 h NO_2_ there was a 0.71% increase in all-cause mortality, 0.88% increase in cardiovascular mortality, and 1.09% increase in respiratory mortality. As for morbidity, for the same increase in NO_2_ concentration, there was an increase in hospital admissions of 0.57% for respiratory and 0.66% for cardiovascular diseases (3). A large study performed on 652 cities showed that an increase of 10 μg/m^3^ over 2 days of PM_10_ was associated with 0.44% increase in all-cause daily mortality, 0.36% increase in cardiovascular and 0.47% increase in respiratory daily mortality. The increases in daily mortality associated with an increase of 10 μg/m^3^ in PM_2.5_ concentration in 2 days were 0.68%, 0,55%, and 0.74%, respectively for all-cause, cardiovascular and respiratory diseases (4). Corroborating these recent results with older studies data (that showed that an increase of 100 μg/m^3^ in PM_10_ concentration determined an estimated increase in daily mortality rate of all cause with 16%) a dose-effect relationship seems plausible (5, 6).

Also, long term exposure to various pollutants can have an even greater impact on mortality. Long-term exposure to PM_2.5_ increased concentrations at the most vs. less polluted levels was associated with a 1.26-fold increase in all-cause and a 1.37-fold increase in cardiopulmonary mortality rates (7). Long-term exposure to NO_2_ even at sub-guidelines levels increases mortality risk by 4.7% (8). A recent large European study on eight cohorts (ESCAPE) with more than 320,000 participants over 19.5 years showed statistically significant associations (with hazard ratios 1.05 to 1.27) between concentrations of eight PM_2.5_ components (Cu, Fe, K, Ni, S, Si, V, and Zn) and natural-cause mortality, the strongest being for Vanadium (9) suggesting a relation between pollution exposure and life expectancy.

The most important and evident impact of air pollution is on lung health as the lungs are the most exposed organs. Chronic lung diseases such as lung cancer, asthma, COPD, and IPF have been proven to have a link to long-term air pollution exposure. Based on the results of the ESCAPE study, IARC designated PM as group 1 carcinogen for humans as long term exposure to PM_10_ showed 1.22 HR for all lung cancers and 1.51 HR for adenocarcinoma (10, 11). For PM_2.5_, the HR for lung cancer was 1.13 (12). Positive associations between lung cancer incidence and PM_2.5_ and PM_10_ exposures were obtained in an UK study with HRs of 1.63 and 1.53, respectively for increases with 5 μg/m^3^ and 10 μg/m^3^ in concentration, respectively, but in this research, there was also a positive NO_2_ exposure association with a HR of 1.13 for an increase of 20 μg/m^3^ in concentration (13). COPD relations with air pollution are more difficult to assess as the most important risk factor for COPD is smoking. Data from the ESCAPE cohort showed no association of pollutants with COPD. Still, there is statistical evidence from the ELAPSE project that there is an excess risk of developing COPD of 17% for long term exposure to an increase with 5μg/m^3^ in PM_2.5_, of 11% to a 10 μg/m^3^ increase in NO_2_ (14). Inorganic non-particulate pollutants such as NO_2_, NO, CO showed an impact on exacerbation risk (15).

## 2 Materials and methods

An analysis of particulate matter (PM_10_ and PM_2.5_) concentrations in Romania and the impact of particulate matter pollution on morbidity caused by respiratory diseases and mortality caused by various pathologies over a period of 15 years (2010–2024) was performed.

### 2.1 Data and data sources

#### 2.1.1 Pollution data

Particulate matter (PM_10_ and PM_2.5_) average yearly concentrations (μg/m^3^) measured by all stations from all 42 counties in Romania were extracted from 2 sources: TEMPO database (16) of the Romanian National Statistics Institute (NSI) (data availability for 2010–2023) and the National Database of Air Quality Monitoring (NDAQM) database (17) of the Romanian Ministry of Environment, Water and Forests (MEWF) and of the Romanian National Agency of Environment Protection (NAEP) (data availability for 2010–2025). As multiple air quality monitoring stations' data (average yearly concentrations) were available for some counties, an average value was calculated and attributed as yearly average concentration for those counties. A database convergence for particulate matter was performed (the data from NSI database was used as base, and missing data was extracted from MEWF database if available). For PM_10_ out of 672 total values, there were 134 missing values (81.06% data availability) and for PM_2.5_ there were 453 missing values (only 32.59% data availability).

#### 2.1.2 Demographic and public health data

Mortality causes data (cardiovascular diseases (CV) and particularly ischemic heart disease (IHD), cancers, respiratory diseases (RD), diabetes mellitus (DM), tuberculosis (TB), and congenital malformations and chromosomal anomalies, all under 27 days, all under 1 year and all under 5 years of age), were extracted from the NSI database (TEMPO). Morbidity data [admissions for asthma, chronic obstructive pulmonary disease (COPD), interstitial lung disease (ILD), lung cancer (LC), and respiratory infections (RI)] were extracted from the disease related groups (DRG) database of the Romanian National Institute of Management of Health Services (NIMHS) (18) and reported per 100,000 population. The resident population every year in every county was extracted from the NSI (TEMPO) database.

### 2.2 Methods

#### 2.2.1 Software used

For initial research and database building, Microsoft excel was used. Python conversion scripts were used to convert pdf, csv, and xls format exported from institutional database into new excel format (xlsx). The excel file was imported in RStudio, and all the data processing for building tables, charts, graphics, and maps and for statistical analysis were performed in RStudio (19).

#### 2.2.2 Statistical data analysis

Descriptive statistics data were computed (average county values, standard deviation, range, median values), and normality Shapiro-Wilk tests were performed. Most data sets had a non-natural distribution (Table 1).

**Table 1:** Descriptive statistics of particulate matter, morbidity and mortality variables with the normality Shapiro-Wilk test results.

Variable	Unit	All years (Full dataset)				
		Mean	SD	Median	Range	Natural distribution
PM _10_	μg/m3	23.13	5.69	22.54	8.7–40.2	No
PM _2.5_	μg/m3	15.94	4.32	15.27	6.9–32.2	No
RI	Adm/100 k	587.2	221.3	557.42	135.1–1,335.5	No
ILD	Adm/100 k	275.7	202.7	215.11	16.2–886.5	No
COPD	Adm/100 k	340.7	187.5	328.96	20.7–1,088.9	No
LC	Adm/100 k	119.3	54.19	113.02	14.5–266.3	No
Asthma	Adm/100 k	93.68	65.71	78.14	5.2–417.5	No
TB	Deaths/100 k	4.49	2.46	4.11	0.3–18.6	No
Cancers	Deaths/100 k	221.8	28.77	219.74	132.8–314.2	Yes
DM	Deaths/100 k	13.44	13.15	10.34	0.2–102.1	No
CV	Deaths/100 k	701.2	146.7	681.62	406.1–1,392.2	No
IHD	Deaths/100 k	235.9	73.08	228.86	71–460.8	No
RD	Deaths/100 k	98.73	67.85	76.07	23.3–439.6	No
Congenital	Deaths/100 k	1.88	1.02	1.72	0.2–6	No
Neonatal (< 27 days)	Deaths/1 k births	4.28	1.8	4	0.8–10.9	No
Infant (< 1 year)	Deaths/1 k births	7.47	2.77	7	2.2–17.3	No
Child (< 5 years)	Deaths/1 k births	9.04	3.13	8.6	2.7–19.6	No

RI, respiratory infections; ILD, interstitial lung diseases; COPD, chronic obstructive pulmonary disease; LC, lung cancers; TB, tuberculosis; Cancers, all cancer types; DM, diabetes mellitus; CV, cardiovascular; IHD, ischemic heart disease; RD, respiratory diseases; Congenital, congenital malformations and chromosomal anomalies.

**Figure 1:**
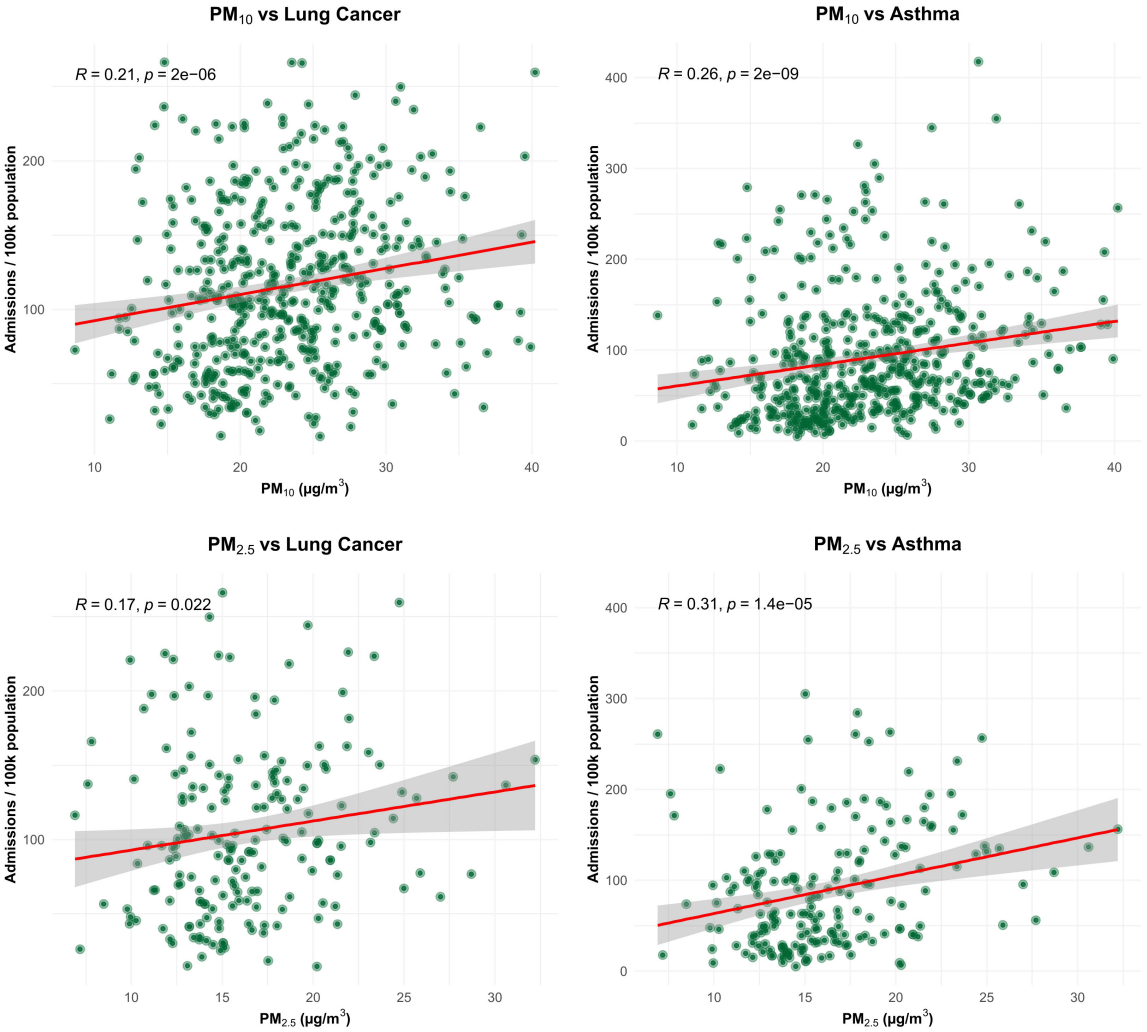
Spearman correlation scatter-dot plots for correlations between PM_10_ and PM_2.5_ concentrations and morbidities for lung cancers and asthma.

**Figure 2:**
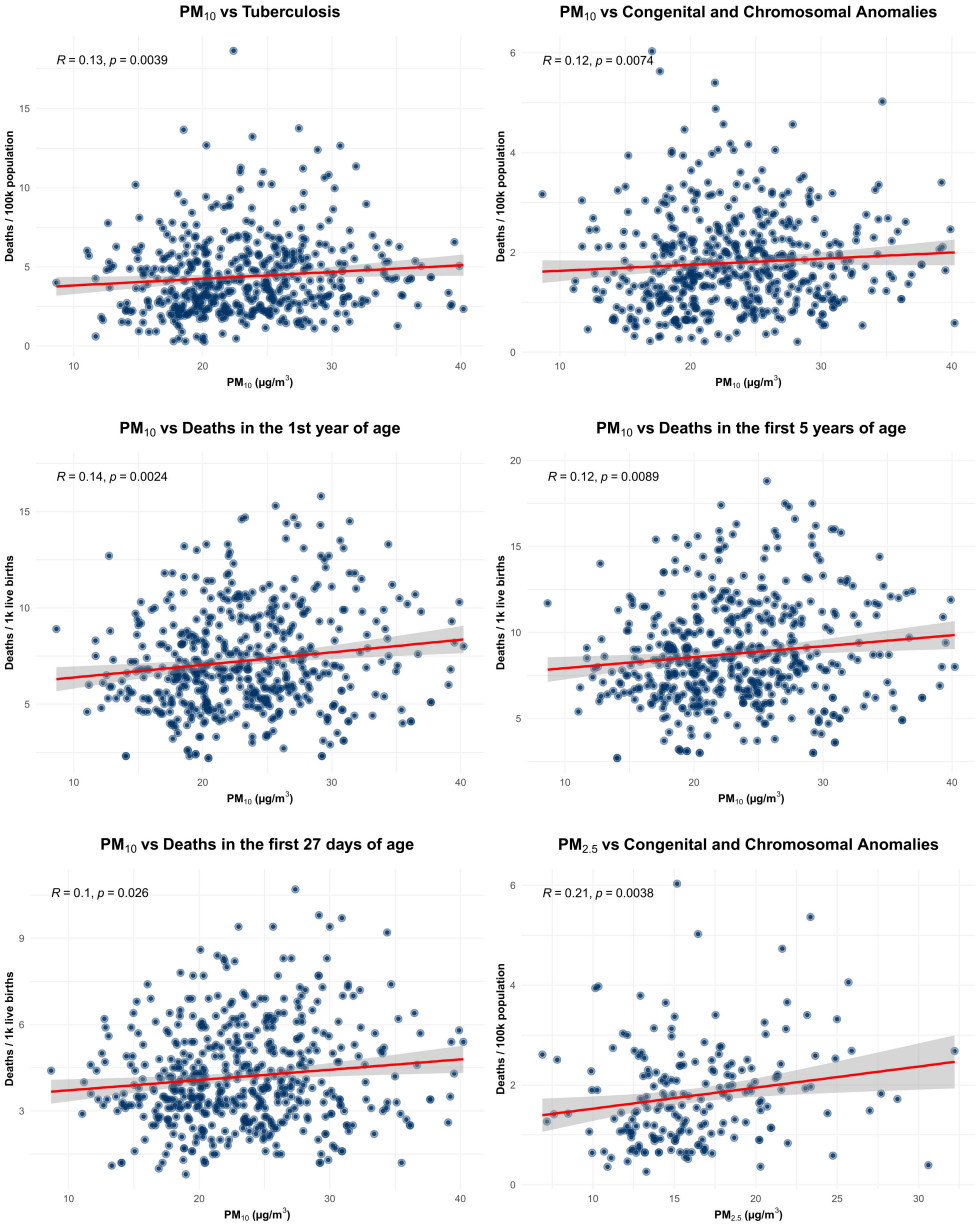
Spearman correlation scatter-dot plots for correlations between PM_10_ and mortalities for tuberculosis, congenital, and chromosomal anomalies, early childhood deaths and between PM_2.5_ and mortality for congenital and chromosomal anomalies.

The correlation tests between contemporaneous (same year) PM average values and morbimortality data were performed using the non-parametric Spearman method with the generation of scatter-dot Spearman correlation plots for the most relevant correlations discovered (Figures 1, 2).

A comparative *t*-test (Welch 2 sample *t*-test) was performed using data from the most polluted and least polluted county in terms of average concentration of particulate matter during the entire period of 15 years.

To estimate the impact of longer exposure to particulate matter on regional morbidity and mortality, a longitudinal regression approach was employed. First, a single pollutant model (simple linear regression—Equation 1) was used to determine the cumulative effect of PM_10_ or PM_2.5_ over 3-years and 5-years moving exposure windows. This way, moving averages were calculated to capture the chronic, cumulative burden of air pollution on hospital admissions and death rates. Secondly, a dual pollutant (multivariate) model (Equation 2) was used using the moving 3-years and 5-years windows. This model simultaneously included fine particles (PM_2.5_) and coarse particles (PM_Coarse_, defined as PM_10_-PM_2.5_) as independent predictors, allowing to assess the independent health risks associated with different particle sizes.


$$\begin{array}{l} Y=A+\beta \times \left(PM_{fraction}\right) \end{array}$$ (1)

$$\begin{array}{l} Y=A+\beta_{2.5}\times \left(PM_{2.5}\right)+\beta_{Coarse}\times \left(PM_{Coarse}\right) \end{array}$$ (2)

The regression analysis results allowed to estimate the avoidable burden of admissions for asthma and lung cancer for the interval of 15 years. The average values for PM_2.5_ and PM_10_ in a certain year were taken into consideration and the no-burden threshold was established at the WHO recommended values of 5 μg/m^3^ for PM_2.5_ and 15 μg/m^3^ for PM_10_. The difference was multiplied by the beta coefficient for morbidities for admissions for asthma and lung cancer to give the avoidable burden for the considered year. The values expressed in cases per 100,000 population were summarized to give cumulative avoidable burden (CAB_c_–Equation 3) for a county in all 15 years (2010–2024).


$$\begin{array}{l} CAB_{c}=\sum_{i=2010}^{i=2024}\beta \times \max\left(0,PM_{c,i}-PM_{Target}\right) \end{array}$$ (3)

Some PM_2.5_ data was missing and for this estimation a filling technique was used consisting of: local ratio calculation (for each county, a historical PM_2.5_/PM_10_ ratio was calculated based on years with concurrent monitoring), ratio application (missing PM_2.5_ values were estimated by applying the local ratio to observed PM_10_ levels), national baseline (in instances where no local PM_2.5_ data was available, a national average ratio of 0.65 was applied as a fallback to maintain physical consistency between particulate fractions) and temporal smoothing (residual short-term gaps ( ≤ 3 years) were resolved using linear interpolation).

#### 2.2.3 Graphics and maps

**Figure 3:**
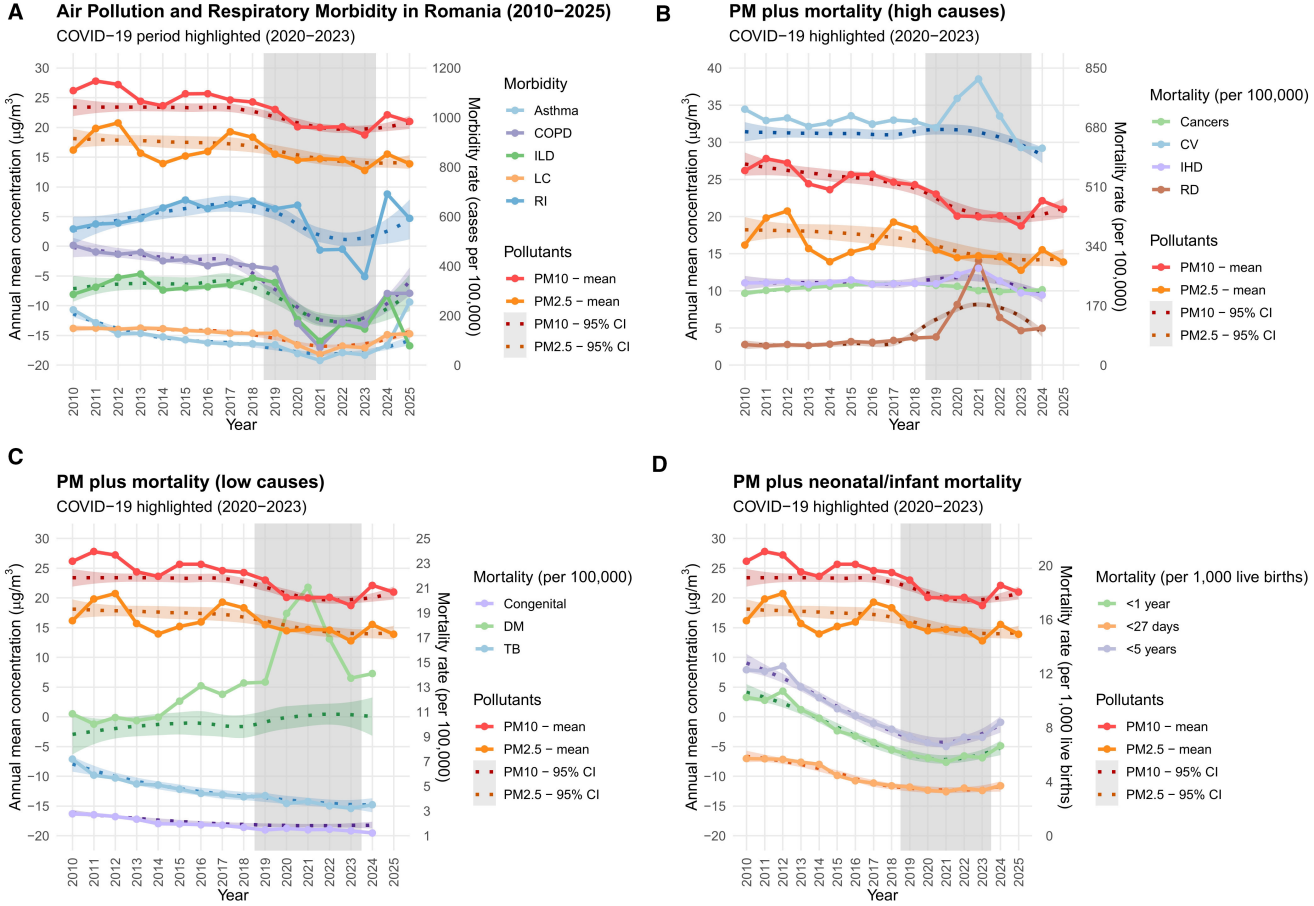
Particulate matter (PM_10_ and PM_2.5_) pollutants mean concentration evolution in Romania during 2010–2025 overlapped with: **(A)** reported respiratory morbidity evolution (admissions per 100,000 population) during 2010–2025; RI, respiratory infections; ILD, interstitial lung diseases; COPD, chronic obstructive pulmonary disease; LC, lung cancers; **(B)** calculated mortality for high mortality causes (CV, cardiovascular ow which OHD, ischemic heart disease, RD, respiratory diseases and all types of cancers); **(C)** calculated mortality for low mortality causes (DM, diabetes mellitus, TB, tuberculosis, and Congenital and chromosomal anomalies); **(D)** childhood mortalities (age < 27 days, age < 1 year, and age < 5 years).

Comparative 15-year evolution graphics were generated in RStudio for the two pollutants and morbidity and mortality indicators with connected lines, trend lines and confidence intervals (Figure 3).

**Figure 4:**
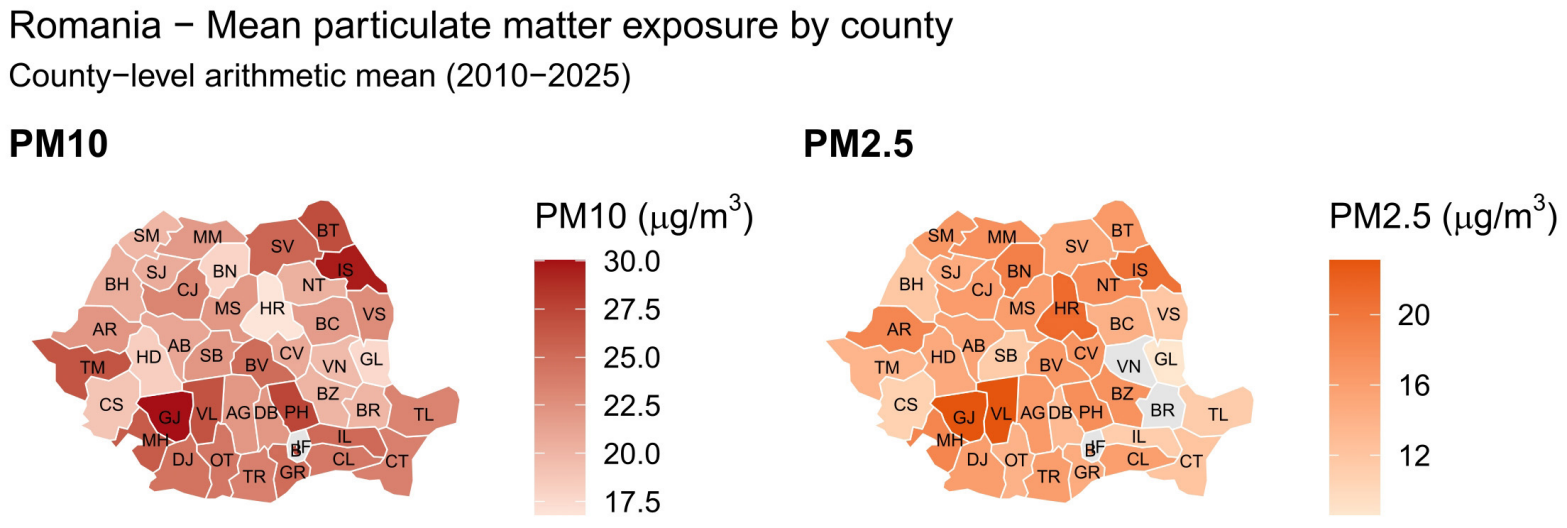
Choropleth maps of PM_10_ and PM_2.5_ mean values during 2010–2024 years in the Romanian counties.

**Figure 5:**
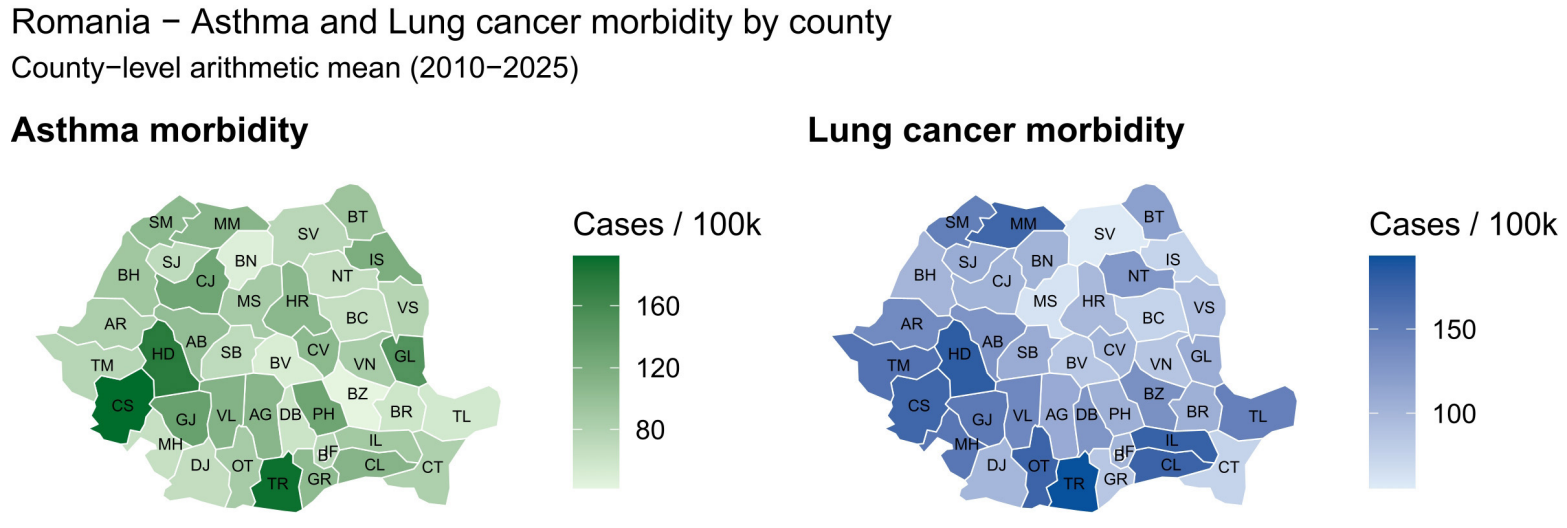
Asthma and lung cancer average morbidity maps.

Pollution, morbidity, and mortality choropleth heatmaps were generated using RStudio (Figure 4). Morbidity and mortality maps were generated for the most relevant pathologies positively correlated with particulate matter pollution (Figure 5).

**Figure 6:**
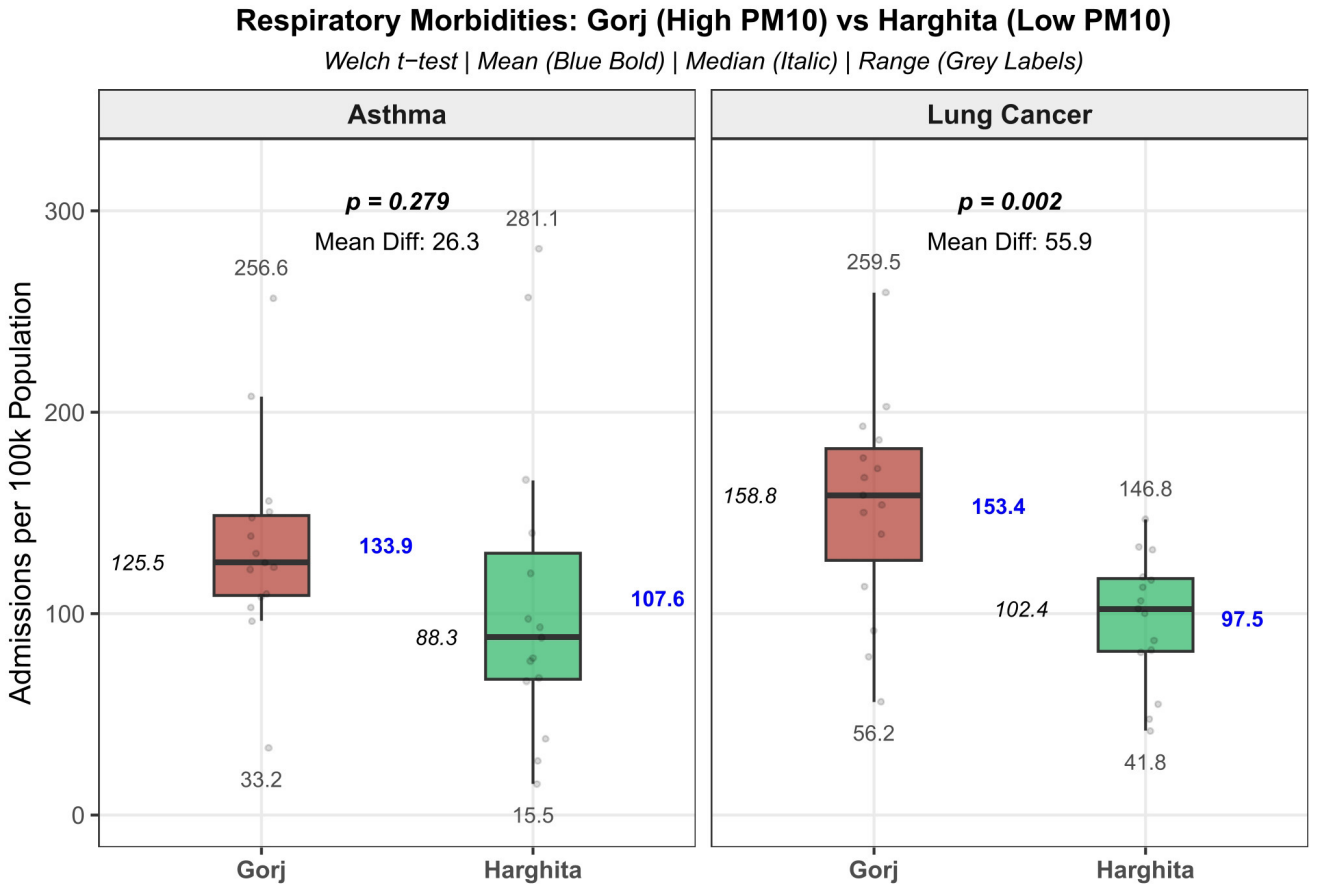
Boxplot chart with results of Welch two samples *t*-test showing differences in morbidities for asthma and lung cancer in most (Gorj) and least (Harghita) PM_10_ polluted counties.

**Figure 7:**
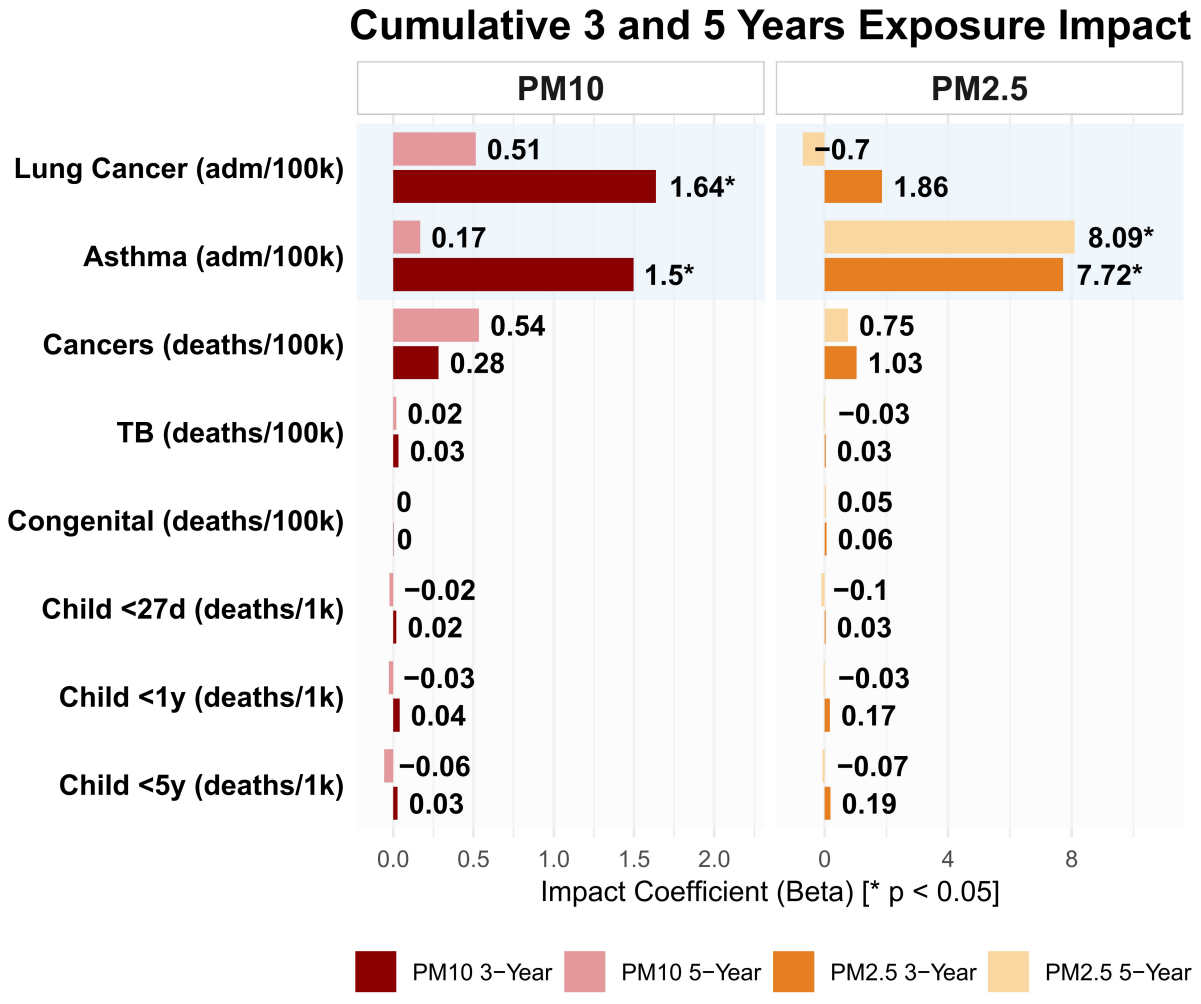
Cumulative exposure impact of PM_10_ and PM_2.5_ on morbidities and mortalities using single pollutant regression model and 3-years and 5-years moving windows.

**Figure 8:**
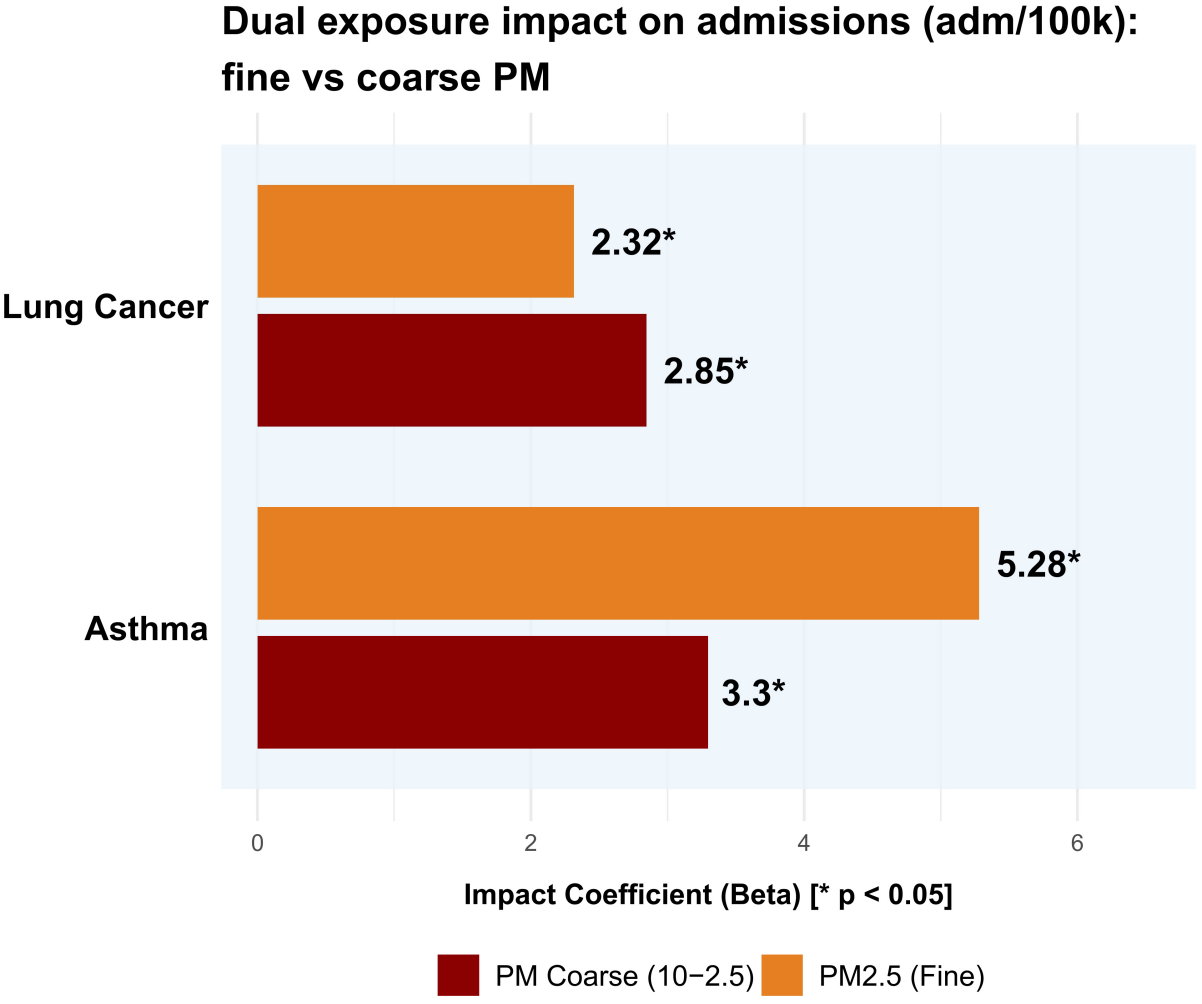
Cumulative dual exposure impact of PM_Coarse_ and PM_2.5_ on morbidities of lung cancer and asthma using dual pollutant regression model and 3-years moving windows.

**Figure 9:**
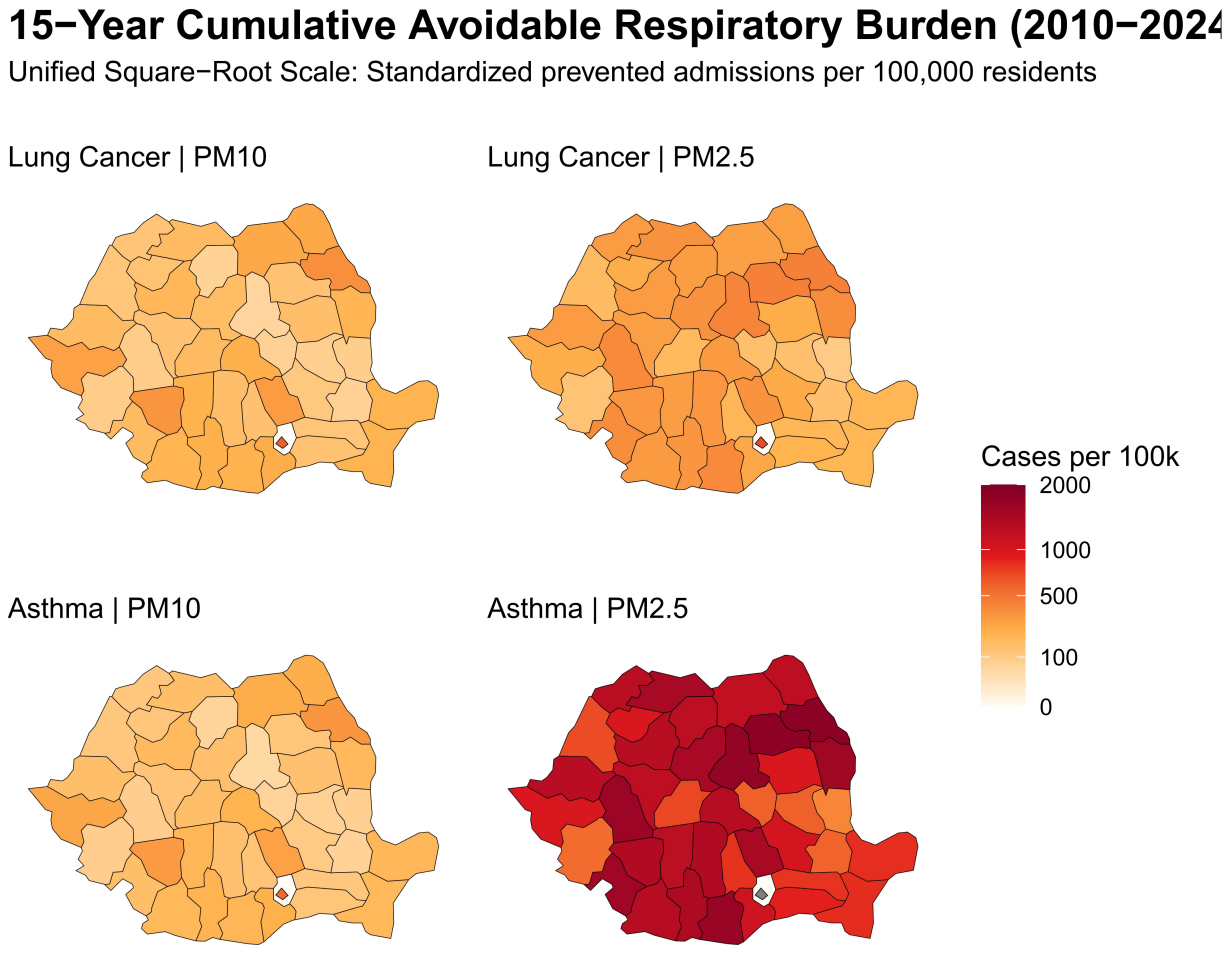
15-Year cumulative avoidable respiratory burden maps for Lung cancer and asthma.

Also, charts related to comparative tests between most and least polluted counties (Figure 6), charts related to cumulative single (Figure 7) and dual exposure (Figure 8) impact and maps of cumulative avoidable burden (Figure 9) were generated with RStudio.

## 3 Results

### 3.1 Pollution and demographic data

The pollution and demographic data are presented in Table 1. Most of the studied variables had a non-natural distribution. Through the studied period, the mean concentration of PM_10_ was 23.13 μg/m^3^ (SD = 5.69) ranging from 8.7 to 40.2 in different counties. The PM_2.5_ had a mean concentration of 15.94 μg/m^3^ (SD = 4.32) ranging from 6.9 to 32.2 based on available data. The particulate matter concentration had a decrease through the years with not so evident impact of SARS-Cov2 (COVID-19) pandemic (Figure 3A).

Pollution heatmaps show areas of increased and decreased pollution by particulate matter (Figure 4). The most polluted counties by PM_10_ were Gorj (GJ), Iasi (IS), and Prahova (PH) and by PM_2.5_ were Vâlcea (VL), Gorj and Harghita (HR). The least polluted counties by PM_10_ were Harghita, Galati (GL), and Bistrita-Năsăud (BN) and by PM_2.5_ were Galati, Caras-Severin (CS), and Ialomita (IL) (Table 2). Gorj county consistently appears as one of the most polluted counties in both categories, likely due to its intensive industrial and mining activities. Galati ranks among the least polluted for both PM_10_ and PM_2.5_ in this dataset. Harghita presents an interesting case: while it has very low PM_10_ levels (the lowest in the country), it appears in the top 3 for PM_2.5_. This discrepancy may be due to the significantly lower number of PM_2.5_ monitoring records compared to PM_10_, or local factors such as residential heating (biomass burning) which contributes more to fine particles—PM_2.5_.

**Table 2:** Most and least polluted counties in Romania by mean concentrations of PM_10_ and PM_2.5_ (μg/m^3^) during 2010–2025 periods based on available data.

Rank	Most PM_10_ polluted counties	Mean PM_10_	Least MP_10_ polluted counties	Mean PM_10_
1	Gorj	30.03	Harghita	16.81
2	Iasi	29.63	Galati	17.52
3	Prahova	27.37	Bistrita-Năsăud	17.92
**Rank**	**Most PM**_2.5_ **polluted counties**	**Mean PM**_2.5_	**Least PM**_2.5_ **polluted counties**	**Mean PM**_2.5_
1	Vâlcea	23.09	Galati	8.61
2	Gorj	23.07	Caras-Severin	10.69
3	Harghita	21.22	Ialomita	11.17

During the years 2020–2022, the SARS-Cov2 (COVID-19) pandemic had a profound impact on reported morbidity and mortality (Figures 3A–D). The least impacted variables seem to be asthma and lung cancer morbidities (admissions per 100,000 population) and mortalities caused by all types of cancers, tuberculosis, and congenital and chromosomal anomalies. Among childhood mortalities, the least impacted seems to be newborn deaths per 1,000 live newborns.

### 3.2 Impact of pollution by particulate matter on respiratory morbidity and mortality

#### 3.2.1 Contemporaneous correlations

Spearman correlation tests were performed to study the correlation between contemporaneous average yearly county concentrations of particulate matter in the air and epidemiologic indicators of morbidity and mortality using available data (Table 3).

**Table 3:** Spearman correlation table for PM_10_ and PM_2.5_ concentrations (μg/m^3^) with morbidity and mortality.

Indicators	Variable	All years			
		PM_10_ statistics		PM_2.5_ statistics	
		*r*	*p*	*r*	*p*
Morbidity/100 k	RI^*^	−0.03	0.51	−0.011	0.876
	ILD^*^	0.139	0.002	0.19	0.009
	COPD^*^	0.293	< 0.001	0.259	< 0.001
	LC	0.211	< 0.001	0.166	0.022
	Asthma	0.264	< 0.001	0.31	< 0.001
Mortality/100 k	TB	0.129	0.004	0.007	0.922
	Cancers	0.061	0.177	0.103	0.157
	DM^*^	−0.087	0.052	0.046	0.535
	CV^*^	−0.092	0.041	0.051	0.486
	IHD^*^	−0.217	< 0.001	0.058	0.43
	RD^*^	−0.414	< 0.001	−0.253	< 0.001
	Congenital	0.12	0.007	0.21	0.004
Mortality/1 k	< 27 days	0.1	0.026	0.091	0.211
	< 1 year	0.135	0.002	0.128	0.079
	< 5 years	0.117	0.009	0.09	0.218

RI, respiratory infections; ILD, interstitial lung diseases; COPD, chronic obstructive pulmonary disease; LC, lung cancers; TB, tuberculosis; Cancers, all cancer types; DM, diabetes mellitus; CV, cardiovascular; IHD, ischemic heart disease; RD, respiratory diseases; Congenital, congenital malformations and chromosomal anomalies; ^*^big influence of COVID years on reported morbidity and mortality–correlation results not reliable (or blue – COVID influenced; green – positive correlation; red – no correlation or no statistical significance).

Due to the high bias induced by reported morbidity and mortality data during the SARS-Cov2 pandemic years (2020–2022), only correlations regarding morbidities for LC and Asthma and mortalities for TB, all types of cancers, congenital and chromosomal anomalies and early childhood can be considered. We can notice a statistically significant weak positive correlation of PM_10_ concentration with morbidities for LC and asthma and very weak with mortalities for TB, congenital and chromosomal anomalies and with childhood deaths (among which deaths < 1 year of age seems most positive). In what concerns PM_2.5_ concentration, there is a statistically significant weak positive correlation with asthma morbidity and congenital and chromosomal anomalies mortality and very weak positive correlation with lung cancer morbidity (Figures 1, 2). According to the data available, there is no correlation between contemporaneous particulate matter concentration and mortality of all cancer types. Tuberculosis and early childhood mortalities showed no correlation with PM_2.5_ concentration. A possible cause may be the inconsistency of available data for PM_2.5_ concentrations. As asthma and lung cancers morbidity has the most consistent correlation with particulate matter pollution morbidity maps were generated (Figure 5).

#### 3.2.2 Most vs. least polluted counties comparison

To better understand the differences in morbimortality data based on PM pollution, a comparative Welch 2 sample *t*-test between most and least PM_10_ polluted counties (Gorj – 30.03 μg/m^3^ and Harghita – 16.80 μg/m^3^, respectively) was performed. The test could not be performed for PM_2.5_ as there is a significant data inconsistency with a high count of missing values. From the Spearman correlation test, there are positive correlations in reported morbidity (admissions /100,000 population) for asthma and lung cancers, and these variables were chosen for the *t*-test (Table 4, Figure 5). A statistically significant disparity in LC admission rates was observed (*p* = 0.002). Gorj exhibited a substantially higher mean admission rate (153.4 ± 52.6 per 100 k) compared to Harghita (97.5 ± 29.5 per 100 k), representing an approximate 57% increase in burden. While Gorj reported a higher mean admission rate for Asthma (133.9 ± 88.3 per 100 k) than Harghita (107.6 ± 38.6 per 100 k), this difference did not reach statistical significance (*p* = 0.279), likely due to the high inter-annual variability observed in the high-pollution cohort.

**Table 4:** *T*-test results for comparison between the county with the highest recorded PM_10_ levels (Gorj, 30.03 μg/m^3^) and the lowest (Harghita, 16.80 ug/m^3^).

Variable	Gorj (High PM_10_)		Harghita (Low PM_10_)		T statistics			
	Mean	SD	Mean	SD	T-statistic	*p-value*	CI lower	CI upper
Asthma	133.85	50.379	107.55	76.863	1.108	0.279	−22.657	75.258
Lung cancer	153.41	52.187	97.523	31.684	3.546	0.002	23.292	88.498

#### 3.2.3 Cumulative exposure impact (single-pollutant model)

With the single linear regression model, the 3-year exposure window emerged as the most robust predictor for morbidity. Lung Cancer admissions showed a significant positive association with 3-year PM_10_ moving average concentration (β = 1.64, *p* = 0.004). A 1 μg/m^3^ increase in the 3-year average concentration of PM_10_ was associated with a statistically significant increase of 1.64 lung cancer admissions per 100,000 inhabitants (*p* = 0.004). Asthma admissions were significantly influenced by PM_2.5_ across both the 3-year (β = 7.73, *p* < 0.001) and 5-year (β = 8.09, *p* = 0.025) windows, indicating a strong, sustained response to fine particulate matter. For every 1 μg/m^3^ increase in the 3-year cumulative exposure to PM_2.5_, an additional 7.73 asthma admissions per 100,000 inhabitants (*p* < 0.001) was observed. The long-term 5-year moving average of PM_2.5_ concentration showed the strongest correlation with asthma morbidity, adding 8.09 admissions per 100,000 inhabitants for each unit of increase (*p* = 0.025) (Table 5, Figure 7).

**Table 5:** β values (with *p-values*) for PM_10_ and PM_2.5_ from single pollutant regression analysis for 3-years and 5-years moving windows cumulative exposure for morbidities and mortalities.

Parameter	PM_10_ 3 years	PM_10_ 5 years	PM_2.5_ 3 years	PM_2.5_ 5 years
Lung cancer (/100 k)	1.64 (0.004)^*^	0.51 (0.455)	1.86 (0.291)	−0.70 (0.801)
Asthma (/100 k)	1.50 (0.011)^*^	0.17 (0.808)	7.73 (< 0.001)^*^	8.09 (0.025)^*^
Cancers (/100 k)	0.28 (0.324)	0.53 (0.130)	1.03 (0.175)	0.75 (0.417)
TB (/100 k)	0.03 (0.134)	0.02 (0.453)	0.03 (0.631)	−0.03 (0.740)
Congenital (/100 k)	0.00 (0.806)	−0.00 (0.968)	0.06 (0.103)	0.05 (0.482)
Child deaths < 27 d (/1 k)	0.02 (0.245)	−0.02 (0.237)	0.03 (0.644)	−0.10 (0.309)
Child deaths < 1 y (/1 k)	0.04 (0.105)	−0.03 (0.295)	0.17 (0.112)	−0.03 (0.851)
Child deaths < 5 y (/1 k)	0.03 (0.315)	−0.06 (0.069)	0.19 (0.137)	−0.07 (0.734)

^*^marks statistically significant results.

#### 3.2.4 Cumulative dual exposure impact

For this purpose, a dual (multivariate) regression model was used using moving average concentrations of PM_10_ and PM_2.5_ of 3-years windows. For asthma morbidity, while both fractions remained significant, PM_2.5_ was the dominant driver (β_2.5_ = 5.28, *p* < 0.001). For lung cancer, the dual-pollutant model “unmasked” the impact of fine particles. While PM_2.5_ was non-significant in the single model, it became statistically significant in the dual model (β_2.5_ = 2.32, *p* = 0.017) after adjusting for coarse dust. The coarse fraction (PM_Coarse_) remained a highly significant independent predictor for lung cancer (β_Coarse_ = 2.85, *p* < 0.001), suggesting that larger industrial particles play a specialized role in oncology admissions. The model calculates the effect of one pollutant while holding the other constant (Table 6, Figure 8). For asthma, in the multivariate model, the fine fraction (PM_2.5_) remained the primary independent driver of asthma morbidity, adding 5.28 admissions per 100,000 inhabitants for every increase with 1 μg/m^3^, independent of coarse particle levels. Simultaneously, the coarse fraction (PM_Coarse_) contributed an independent increase of 3.30 asthma admissions per 100,000 inhabitants for every unit increase in concentration. For lung cancer, the coarse particle fraction exhibited the strongest independent association with oncology hospitalizations, adding 2.85 lung cancer admissions per 100,000 inhabitants for every unit increase. After adjusting for dual-exposure model, the impact of the fine fraction (PM_2.5_) exposure for 3 years on lung cancer morbidity was unmasked and became statistically significant, adding 2.32 admissions per 100,000 inhabitants for every unit increase in concentration.

**Table 6:** β values (with *p-values*) for PM_10_ and PM_2.5_ from single and dual pollutant regression analysis for 3-years and 5-years moving windows cumulative exposure for morbidities for lung cancer and asthma.

Health outcome (admissions /100,000 population)	Window	Pollutant	Single β (*p*)	Dual β (*p*)
Lung cancer	3-Year	PM_2.5_ (Fine)	1.86 (0.291)	2.32 (0.017)^*^
		PM_Coarse_	2.85 (< 0.001)^*^	2.85 (< 0.001)^*^
	5-Year	PM_2.5_ (Fine)	−0.70 (0.801)	−0.21 (0.910)
		PM_Coarse_	1.21 (0.045)^*^	1.21 (0.045)^*^
Asthma	3-Year	PM_2.5_ (Fine)	7.73 (< 0.001)^*^	5.28 (< 0.001) ^*^
		PM_Coarse_	3.30 (< 0.001)^*^	3.30 (< 0.001)^*^
	5-Year	PM_2.5_ (Fine)	8.09 (0.025)^*^	6.15 (0.041)^*^
		PM_Coarse_	0.81 (0.420)	0.81 (0.420)

^*^marks statistically significant results.

#### 3.2.5 Cumulative avoidable burden

The statistical analysis results can help in tailoring public health policies. For this purpose of acknowledging the effects of pollution at the country level emphasizing the high-burden regions, cumulative avoidable burden maps were constructed. Using the linear regression analysis results [beta coefficients of 1.636 (PM_10_, LC), 1.863 (PM_2.5_, LC), 1.498 (PM_10_, Asthma), and 7.725 (PM_2.5_, Asthma)], and calculating the yearly avoidable admissions for asthma and lung cancer if the concentrations of PM_10_ and PM_2.5_ would be reduced to safe levels (endorsed by WHO), it was possible to build 15-years cumulative avoidable admissions/100k population maps (Figure 9). While reduction in PM_10_ and PM_2.5_ to safe levels would avoid 100 to 500 lung cancer admissions/100 k in 15 years (a similar result also for asthma admissions in the case of lowering PM_10_ concentrations), for asthma a reduction of PM_2.5_ under 5 μg/m^3^ would significantly reduce morbidity by avoiding 1,000 to 2,000 admissions /100 k. These maps can be used by the authorities for communication and easier identification of problematic regions that may require rapid intervention. Also, these maps can be used for population education and increase in awareness of pollution and its impact on public health.

## 4 Discussion

### 4.1 Study limitations

One study limitation is related to missing data of particulate matter average of yearly measured concentrations for some counties and years from the public institutions databases. The most affected dataset is for the PM_2.5_ concentrations. Although the intervention on the data with data-filling techniques was avoided to not influence statistical results, such methods were used to build maps to estimate the avoidable burden, considering that such techniques are more suitable for estimative approaches rather than for statistical analysis. Also, it should be noted that the data related to the two pollutants analyzed present a certain degree of inhomogeneity, because in some counties, the number of air quality monitoring stations is higher than in other counties. This limitation made it impossible for a reliable analysis of differences between the most and least PM_2.5_ polluted counties and may have an impact on statistical significancy of the correlations with public health variables.

The other study limitation is related to the high influence of the SARS-Cov2 (COVID-19) pandemic on recording morbidity and mortality data mostly during the years 2020–2022. The impact of COVID pandemic on respiratory morbidity data is most likely caused by the destination change of most respiratory care units to care for COVID patients so access to respiratory care hospitals was limited for other respiratory pathology patients. These patients were mostly redirected to outpatient care. For mortality data, the shift is reversed toward a spike in reported deaths for cardiovascular, respiratory and diabetes complications as most COVID deaths occurred in patients with COVID-19 pneumonia and these comorbidities. This limitation made practically impossible the study of correlation between the particulate matter concentration and some respiratory morbidity causes (interstitial lung diseases, COPD, respiratory infections) and mortality causes (diabetes mellitus, cardiovascular diseases, respiratory diseases).

Another limitation is not taking into account urbanization. A limitation of the study on urban resident population was taken into account, as most of the measurement stations are positioned in urban areas. Still, disregarding in its entirety the rural population is not recommended as there is a continuous migration of the population between the two environments. People that live in rural areas work in urban areas and vice-versa.

### 4.2 Findings and conclusions

Figure 3 shows a trend in decrease of aggregated yearly average PM_2.5_ and PM_10_ concentrations values at the country level. This occurred most probably as a consequence of implementing technologies of green energy generation and policies of environmental protection through the years.

The study found a credible, statistically significant weak positive correlation between contemporaneous particulate matter concentration (PM_10_ and PM_2.5_) and asthma and lung cancer morbidities (admissions per 100,000 population). Also, PM_10_ concentration is found to correlate with some of the studied mortality causes (tuberculosis, congenital and chromosomal anomalies, and early childhood). This result is expected as other studies cited in the present paper showed an influence of even very short-term exposure on increased particulate matter concentrations may influence daily all-causes of deaths. Still, contemporaneous pollution, from a physio pathological point of view, is more expected to correlate with morbidity rather than mortality, as mortality from a chronic disease is related to the development of the disease on a long period of time in which pollution exposure may play a role. The influence of PM_10_ and PM_2.5_ measured in the same year on congenital and chromosomal anomalies mortality is explainable as PM_2.5_ is demonstrated to be able to enter in the small airways and alveoli and even in the blood stream carrying substances with mutagenic effects (20).

There is a geospatial partial superposition of the choropleth maps of particulate matter pollution and morbidity by asthma and lung cancers. The most PM_10_ polluted county is Gorj and the least polluted is Harghita and the statistically significant difference between morbidity of lung cancers between these counties show that PM_10_ exposure may play a role in the risk of developing lung cancer. Although not statistically significant, the difference in morbidities from asthma between the two counties shows a trend that suggests that PM_10_ may still play a role also in asthma admissions (mostly exacerbations) and lack of control.

This study demonstrates that cumulative 3-year exposure is a critical determinant of regional respiratory morbidity. We conclude that PM_2.5_ is the primary driver of asthma admissions while both fine and coarse particles contribute independently to lung cancer risk. The results confirm a “toxic debt” phenomenon, where hospital admissions for chronic respiratory diseases are more closely tied to cumulative pollution from the preceding 3 years than to acute annual peaks.

As a result of the dual pollutant regression analysis, physio pathologically, the high sensitivity of asthma to PM_2.5_ (β_2.5_ **=** 5.28) supports the hypothesis and scientific literature evidence that fine combustion particles penetrate deep into the lower respiratory tract, triggering acute inflammation (21) and carcinogenic effects (22). Conversely, the robust signal from PM_Coarse_ in lung cancer (β_Coarse_ = 2.85) points to the significant hazard posed by larger industrial and mechanical dust particles which deposit in the primary bronchi and contribute to localized cellular damage (22).

The results of the statistical longitudinal data analysis can be used to build useful avoidable burden maps based on which policies for increasing awareness and for applying green technologies interventions in most affected regions can be tailored. Policy interventions must target both combustion-related fine particles and industrial coarse dust to effectively reduce the long-term healthcare burden in industrial regions. The policies implemented in Romania in during 2010–2024 were effective in decreasing both PM pollutant levels.

## Data Availability

The data used for the contributions presented in the study are included in the article's supplementary material. This data was extracted from the publicly available databases (16–18). Further inquiries can be directed to the corresponding authors.

## References

[1] Tran HM Tsai FJ Lee YL Chang JH Chang LT Chang TY . The impact of air pollution on respiratory diseases in an era of climate change: a review of the current evidence. Sci Total Environ. (2023) 10:898. doi: 10.1016/j.scitotenv.2023.166340 37591374

[2] Schwartz J Fong K Zanobetti A. A national multicity analysis of the causal effect of local pollution, NO_2_, and PM_2.5_ on mortality. Environ Health Perspect. (2018) 126:087004. doi: 10.1289/EHP2732 30235421 PMC6375387

[3] Mills IC Atkinson RW Kang S Walton H Anderson HR. Quantitative systematic review of the associations between short-term exposure to nitrogen dioxide and mortality and hospital admissions. BMJ Open. (2015) 5:e006946. doi: 10.1136/bmjopen-2014-006946 PMC4452753 25967992

[4] Liu C Chen R Sera F Vicedo-Cabrera AM Guo Y Tong S . Ambient particulate air pollution and daily mortality in 652 cities. N Engl J Med. (2019) 381:705–15. doi: 10.1056/NEJMc1913285 31433918 PMC7891185

[5] Pope CA Ransom MR Schwartz J. Daily mortality and PM10 pollution in Utah Valley. Arch Environ Health. (1992) 47:211–7. doi: 10.1080/00039896.1992.9938351 1596104

[6] Dockery DW Schwartz J Spengler JD. Air pollution and daily mortality: Associations with particulates and acid aerosols. Environ Res. (1992) 59:362–73. doi: 10.1016/S0013-9351(05)80042-8 1464289

[7] Dockery DW Pope CA Xu X Spengler JD Ware JH Fay ME . An association between air pollution and mortality in Six U.S. Cities. N Engl J Med. (1993) 329:1753–9. doi: 10.1056/NEJM199312093292401 8179653

[8] Qian Y Li H Rosenberg A Li Q Sarnat J Papatheodorou S . Long-term exposure to low-level NO_2_ and mortality among the elderly population in the Southeastern United States. Environ Health Perspect. (2021) 129. doi: 10.1289/EHP9044 PMC8713651 34962424

[9] Chen J Rodopoulou S de Hoogh K Strak M Andersen ZJ Atkinson R . Long-term exposure to fine particle elemental components and natural and cause-specific mortality-a pooled analysis of Eight European Cohorts within the ELAPSE Project. Environ Health Perspect. (2021) 129. doi: 10.1289/EHP8368 PMC8041432 33844598

[10] Loomis D Grosse Y Lauby-Secretan B El Ghissassi F Bouvard V Benbrahim-Tallaa L . The carcinogenicity of outdoor air pollution. Lancet Oncol. (2013) 14:1262–3. doi: 10.1016/S1470-2045(13)70487-X 25035875

[11] Raaschou-Nielsen O Andersen ZJ Beelen R Samoli E Stafoggia M Weinmayr G . Air pollution and lung cancer incidence in 17 European cohorts: prospective analyses from the European Study of Cohorts for Air Pollution Effects (ESCAPE). Lancet Oncol. (2013) 14:813–22. doi: 10.1016/S1470-2045(13)70279-1 23849838

[12] Hvidtfeldt UA Severi G Andersen ZJ Atkinson R Bauwelinck M Bellander T al. Long et -term low-level ambient air pollution exposure and risk of lung cancer – A pooled analysis of 7 European cohorts. Environ Int. (2021) 146:106249. doi: 10.1016/j.envint.2020.106249 33197787

[13] Huang Y Zhu M Ji M Fan J Xie J Wei X . Air pollution, genetic factors, and the risk of lung cancer: a prospective study in the UK Biobank. Am J Respir Crit Care Med. (2021) 204:817–25. doi: 10.1164/rccm.202011-4063OC 34252012

[14] Liu S Jørgensen JT Ljungman P Pershagen G Bellander T Leander K . Long-term exposure to low-level air pollution and incidence of chronic obstructive pulmonary disease: the ELAPSE project. Environ Int. (2021) 1:146. doi: 10.1289/isee.2020.virtual.P-1211 33276316

[15] Evangelopoulos D Chatzidiakou L Walton H Katsouyanni K Kelly FJ Quint JK . Personal exposure to air pollution and respiratory health of COPD patients in London. Eur Respir J. (2021) 58:2003432. doi: 10.1183/13993003.03432-2020 33542053 PMC8290182

[16] TEMPO Online. (2026). Available online at: http://statistici.insse.ro:8077/tempo-online/#/pages/tables/insse-table (Accessed February 28, 2026).

[17] Reteaua Nationala de Monitorizare a Calitatii Aerului (2026). Available online at: https://calitateaer.ro/public/monitoring-page/reports-reports-page/?__locale=ro (Accessed February 28, 2026).

[18] DRG - Centrul de Cercetare si Evaluare a Serviciilor de Sanatate (2026). Available online at: https://drg.ro/index.php?p=indicatori (Accessed February 28, 2026).

[19] Core Team VA. R: A Language and Environment for Statistical Computing. Vienna, Austria: R Foundation for Statistical Computing (2025). Available online at: https://www.R-project.org (Accessed February 28, 2026).

[20] Zani C Donato F Ceretti E Pedrazzani R Zerbini I Gelatti U . Genotoxic activity of particulate matter and in vivo tests in children exposed to air pollution. Int J Environ Res Public Health. (2021)18:5345. doi: 10.3390/ijerph18105345 PMC8156021 34067860

[21] Luo J Liu H Hua S Song L. The correlation of PM2. 5 exposure with acute attack and steroid sensitivity in asthma. Biomed Res Int. (2022) 2022:2756147. doi: 10.1155/2022/2756147 36033576 PMC9410784

[22] Vu J Nadeau K Kasowski M. Molecular mechanisms of air pollution–induced carcinogenesis and the emerging role of microplastics. Hum Genomics. (2025) 20:6. doi: 10.1186/s40246-025-00880-0 41345682 PMC12781297

